# Towards a Remote Patient Monitoring Platform for Comprehensive Risk Evaluations for People with Diabetic Foot Ulcers

**DOI:** 10.3390/s24102979

**Published:** 2024-05-08

**Authors:** Gozde Cay, M.G. Finco, Jason Garcia, Jill L. McNitt-Gray, David G. Armstrong, Bijan Najafi

**Affiliations:** 1 Digital Health and Access Center (DiHAC), Michael E. DeBakey Department of Surgery, Baylor College of Medicine, Houston, TX 77030, USA; gozde.cay@bcm.edu (G.C.);; 2 Southwestern Academic Limb Salvage Alliance (SALSA), Department of Surgery, Keck School of Medicine of University of Southern California, Los Angeles, CA 90033, USA; 3 Department of Biological Sciences, Dornsife College of Letters, Arts and Sciences, University of Southern California, Los Angeles, CA 90007, USA; 4 Department of Biomedical Engineering, Viterbi School of Engineering, University of Southern California, Los Angeles, CA 90007, USA

**Keywords:** smart offloading, diabetes, diabetic foot ulcer, telemedicine, digital health, remote patient monitoring, personalized care

## Abstract

Diabetic foot ulcers (DFUs) significantly affect the lives of patients and increase the risk of hospital stays and amputation. We suggest a remote monitoring platform for better DFU care. This system uses digital health metrics (scaled from 0 to 10, where higher scores indicate a greater risk of slow healing) to provide a comprehensive overview through a visual interface. The platform features smart offloading devices that capture behavioral metrics such as offloading adherence, daily steps, and cadence. Coupled with remotely measurable frailty and phenotypic metrics, it offers an in-depth patient profile. Additional demographic data, characteristics of the wound, and clinical parameters, such as cognitive function, were integrated, contributing to a comprehensive risk factor profile. We evaluated the feasibility of this platform with 124 DFU patients over 12 weeks; 39% experienced unfavorable outcomes such as dropout, adverse events, or non-healing. Digital biomarkers were benchmarked (0–10); categorized as low, medium, and high risk for unfavorable outcomes; and visually represented using color-coded radar plots. The initial results of the case reports illustrate the value of this holistic visualization to pinpoint the underlying risk factors for unfavorable outcomes, including a high number of steps, poor adherence, and cognitive impairment. Although future studies are needed to validate the effectiveness of this visualization in personalizing care and improving wound outcomes, early results in identifying risk factors for unfavorable outcomes are promising.

## 1. Introduction

Diabetic foot ulcers (DFUs) pose a significant challenge in the management of diabetes and represent a serious complication that requires careful attention. Every 1.2 s, someone develops a DFU and every 20 s, someone with DFU undergoes an amputation [1]. One of the key approaches to treatment for DFUs involves the use of offloading devices, which aim to distribute force and reduce pressure on the wound, thereby facilitating the healing process [2]. While adhering to the appropriate use of these devices is recognized as a crucial strategy to promote the healing of DFUs, some individuals who adhere to offloading device protocols still struggle to achieve healing. This suggests the existence of other contributing factors that need to be considered, including the characteristics of the wound and diabetes, motor function, and patient-reported outcomes [3,4,5]. Although current advancements in DFU management have begun implementing smart technology and wearables, studies have mostly focused on one parameter such as pressure or temperature and informing the patients about the changes in these parameters [6,7,8,9,10,11,12,13].

To distinguish between patients who experience successful healing and those who do not, it is crucial to evaluate and analyze various factors beyond adherence to the device. Understanding the underlying mechanisms associated with healing outcomes can enable healthcare professionals to provide personalized care, leading to more efficient and effective wound treatment. Thus, the objective of this study was to develop a comprehensive visualization tool capable of measuring digital biomarkers closely associated with unsuccessful healing and subsequent visualization for care providers.

Adopting a holistic and personalized technological strategy involves rethinking chronic care to emphasize comprehensive knowledge-building for patients and their care providers. This strategy leverages current advancements in real-time wearable technologies, aligns with electronic health records, and integrates patient-reported outcomes to facilitate real-time, multidimensional data analyses. Current risk-prediction models for the management of DFUs often fall short because they rely on isolated datasets, which fail to capture the full complexity of the condition. Prior studies suggested that visualizing multidimensional data in the management of chronic conditions enhances the understanding of major risk factors, improves patient engagement, and enhances the treatment’s efficacy [14,15,16,17]. With the conversion of complex health data into understandable visuals, both patients and healthcare providers may better discern the patterns and correlations, leading to personalized education for patients and potentially improved patient engagement and tailored treatments [18]. Visual tools support predictive insights for pre-emptive care adjustments and facilitate collaborative care through easy sharing among healthcare teams [19]. Remote patient monitoring (RPM) solutions, initiated by the prolific uptake of smartphones, mobile tablets, and cloud backend services, in tandem with expanding wireless networks and interoperable medical devices, create new opportunities for developing a remote patient monitoring portal as an access point to health information [20,21,22]. This could enable healthcare providers to evaluate various potential risk factors that may impact the outcomes of wound healing, personalize interventions, and enhance patient education, which, in turn, may improve the outcomes of wound healing. The clinical benefit of RPM is consistently observed across diverse populations, even after adjusting for common social determinants of health such as socioeconomic status, access to healthcare, education levels, and geographic location [23]. This suggests that RPM not only compensates for these disparities but also provides a significant opportunity to improve health equity.

However, despite advancements, there remains a significant gap in the design of holistic approaches that integrate all the major risk factors associated with poor wound healing in patients with DFU. Most existing systems fail to adequately simplify and integrate complex data into a format that is readily accessible and actionable for both patients and healthcare providers. This limitation hampers their ability to make informed decisions quickly and effectively. To address this issue, our study proposes the development of a comprehensive graphical interface that aggregates most of the measurable and relevant risk factors into a single, coherent visual representation. This interface aims to display information in an easily understandable manner, facilitating quick assessments and interventions based on a patient’s current health status (e.g., glycemic control, frailty, and cognitive function), behavioral metrics (e.g., adherence to offloading), functional metrics (e.g., daily step counts, balance), and historical data. Additionally, by enabling healthcare providers to view these integrated risk factors at a glance, we expect that the tool will foster more personalized and timely adjustments to care plans, thereby potentially enhancing the outcomes for patients with DFU. Our approach also includes feedback mechanisms that allow for continuous improvement of the tool based on the users’ interactions and its effectiveness in clinical scenarios. Through this study, we aimed to bridge the existing gap and provide a robust solution that leverages technology for the better management of chronic conditions. 

By designing such a holistic visualization tool, our research team hypothesized that clinicians would gain valuable insights into how multiple factors contribute to the healing of DFUs. This tool has the potential to empower healthcare professionals to become more aware of the interacting factors that affect clinical outcomes, as they will be able to identify and monitor specific digital biomarkers associated with improved outcomes of healing. Ultimately, the implementation of this tool could lead to more efficient wound treatment and enhanced patient outcomes in the management of DFU. To our knowledge, this is the first study which combines digital biomarkers and offers a holistic view of the digital biomarkers associated with the outcomes of healing.

## 2. Materials and Methods

### 2.1. Obtaining Digital Biomarkers Associated with Wound Healing

**Figure 1:**
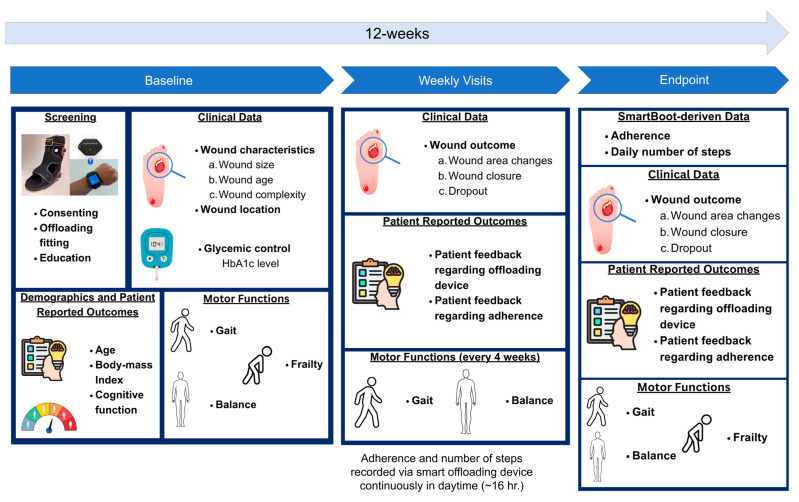
Overall study design.

This manuscript presents preliminary findings from an ongoing parallel randomized controlled trial (ClinicalTrials.gov identifier: NCT04460573) conducted at the Keck School of Medicine, University of Southern California. The overarching goal of the parent study was to investigate how interactive offloading devices, called smart offloading, can improve adherence, and enhance the outcomes of wound healing in individuals with diabetes. The validity and acceptability of the smart boot for the real-time estimation of adherence to offloading and step count have been presented in prior studies [24,25]. However, this study focused on designing and evaluating a holistic remote patient monitoring system. This system aims to simplify the visualization of the key risk factors associated with unfavorable outcomes of wound healing, aiding clinicians in personalizing wound care. It includes tailored patient education that may optimize the outcomes of healing while guiding patients in maintaining healthy mobility and mitigating secondary consequences, such as frailty, poor balance, and gait. Participants meeting specific inclusion criteria, such as having diabetes and a diabetic foot wound, were enrolled in the study, whereas those with high A1c levels (>12), a lack of mobility, multiple wounds, or poor adherence to offloading devices were excluded. The study protocol was approved by the institutional review board of the University of Southern California. Figure 1 illustrates the overall study design. 

Over a period of 12 weeks, participants attended weekly in-person visits, during which, photographs of their ulcer were taken with a wound monitoring device (eKare, eKare Inc., Fairfax, VA, USA) to measure the wound’s dimensions, and their feedback regarding the offloading device and its usage was recorded. Baseline data for each visit on the wound’s complexity and A1c levels were collected from the participants’ medical charts. Additionally, participants underwent a 20 s repetitive elbow flexion–extension test while sitting to assess the upper extremities’ frailty [26,27,28,29,30]. However, instead of using a wrist sensor to assess frailty, we used an alternative solution to extract the kinematics of the elbow’s motion and then estimated the frailty index and frailty phenotypes such as exhaustion, weakness, and slowness using video analysis [31,32]. In addition, to determine their cognitive level, a 12-point Montreal Cognitive Assessment (MoCA) questionnaire was administered by the study coordinator, and a score of 10 points was used as the threshold to determine whether a participant had cognitive impairment [33]. 

At four-week intervals, wearable inertial measurement unit (IMU) sensors developed by BioSensics (LegSys + BalanSense, BioSensics, Newton, MA, USA) were used to measure the participants’ mobility performance, including assessments of gait and balance [34,35,36,37,38,39,40,41,42,43]. The assessment of gait consisted of four walking tests: (1) walking at normal speed (walking 15 feet unassisted at their normal walking speed), (2) walking at fast speed (walking 15 feet unassisted at a safe rapid walking speed), (3) walking at normal speed while counting backwards, and (4) walking 15 feet unassisted at their normal walking speed while counting backwards out loud from the number given by the study coordinator; as well as a timed up and go (TUG) test, starting in a seated position, getting up, walking to the stop sign, turning around, walking back to the chair, and sitting down. The balance assessment consisted of four balance tests: a single stance with eyes open, a single stance with eyes closed, double stances with eyes open, and double stances with eyes closed. For the single-stance–eyes open assessment, the patients stood on one leg unassisted and placed their arms on their hips. For the single-stance–eyes closed assessment, they performed the same position with their eyes closed. During the double-stance–eyes open assessment, the participants stood facing the wall with their feet as close together as possible without touching and with their arms folded across their chests. For the double-stance–eyes closed assessment, the participants stood in the same position, but their eyes were closed. The assessments each took 30 s.

**Figure 2:**
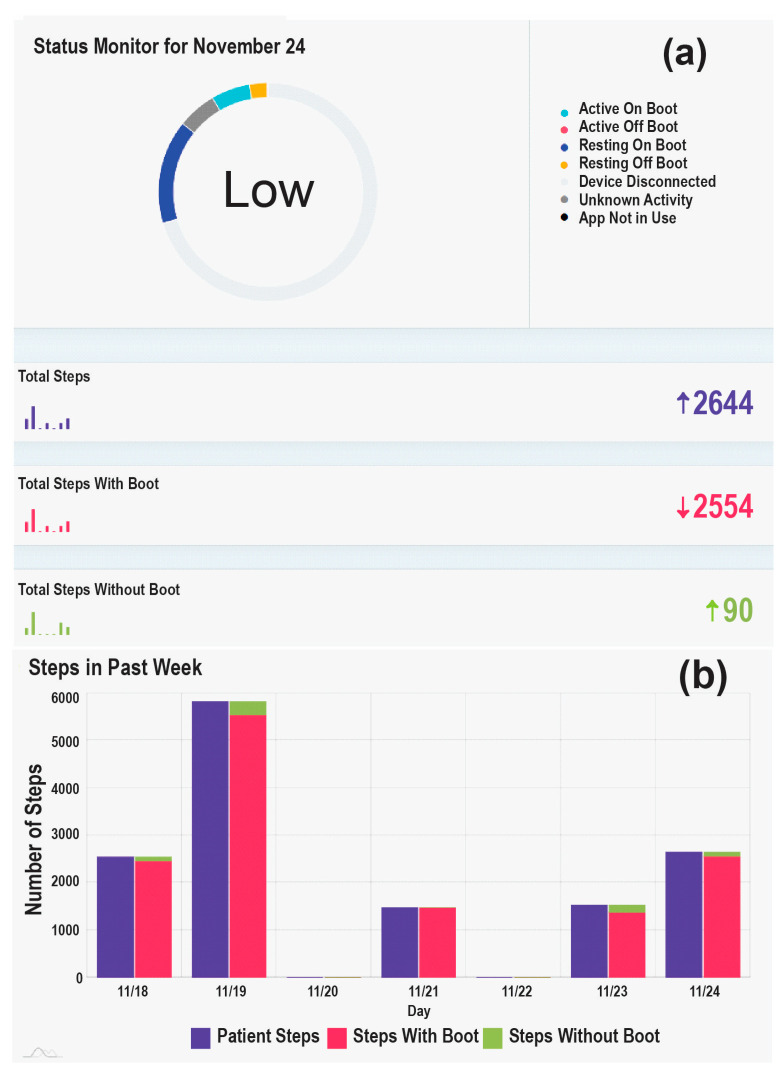
The clinicians’ web dashboard (Sensoria Cloud), designed to empower care providers by enabling remote assessment of adherence levels to offloading and physical activity (PA). (**a**) Daily summary of the offloading device’s activity and number of steps, (**b**) Weekly summary of the number of steps.

To monitor adherence to usage of the offloading device and collect step count data, a smart offloading boot system was used. This system consisted of a removable offloading boot (Foot Defender, Defender Ops., South Miami, FL, USA), a Sensoria Core microelectronics device, and an Android 4G/LTE smartwatch custom app developed by Sensoria Health Inc. (Sensoria Core, Sensoria Health Inc., Redmond, WA, USA) [24,25]. The Sensoria Core utilized an IMU with 6 degrees of freedom to capture the boot’s movements and a Bluetooth low-energy (BLE) module to communicate with the smartwatch. The Sensoria Core transmits the collected data to the smartwatch, which processes and displays real-time information, including the boot’s condition (worn or not worn), activity status (active or resting), step count, and notifications. These data are also sent to the Sensoria cloud system. This enables clinical providers to evaluate the patients’ adherence to offloading and visualize step counts with and without offloading, as shown in Figure 2. Adherence to the offloading boot was calculated using embedded algorithms on the cloud and presented as graphics to the clinical team. Additionally, the website allowed the recording of notifications and alerted the clinician team if the participants removed their boots.

### 2.2. Holistic Visualization to Empower Physicians and Patients

**Figure 3:**
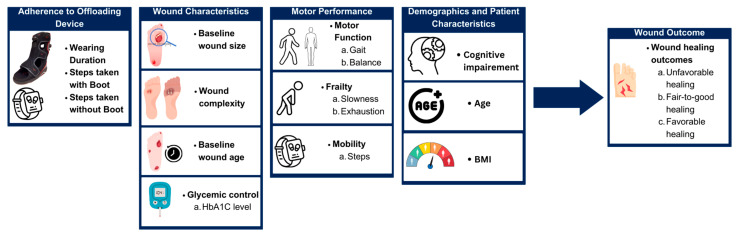
Digital parameters which affect the healing of the diabetic wound.

To effectively visualize the healing trajectory of the patients, we selected various parameters believed to affect the outcomes of wound healing. These parameters included demographics and digital metrics including the wound’s characteristics, frailty phenotypes, gait (cadence), balance, and Smart-Boot-derived digital metrics such as steps and adherence. For frailty phenotypes, we used metrics extracted from the video-based 20 s repetitive elbow flexion–extension test and selected phenotypes that were previously shown to be associated with wound healing, including exhaustion and slowness [44,45]. The details of these parameters, their definitions, and measurement methods are shown in Figure 3 and Table 1.

To simplify interpretation and visualization of the key digital metrics associated with poor wound healing on a single graph, we normalized various parameters believed to affect the outcomes of wound healing on a scale from 0 to 10, where higher values indicated increased risk. For normalization of these parameters, we categorized each metric as low risk (0), medium risk (5), and high risk (10) using standard benchmarks reported in literature; if the standard benchmarks were not reported in the literature, we used the cohort and percentile approach to determine the low-, medium-, and high-risk groups. 

For continuous variables, if the scale was linear and in a limited range (e.g., the MoCA score), then we mapped them to 0 to 10; if needed, we reversed it to indicate that higher values signified higher risk. If the continuous values were not confined within a range and/or the increase in risk did not necessarily have a linear association with these metrics (e.g., age, BMI, cadence, balance, and the wound’s characteristics), then we categorized the variables a low risk (value of 0), medium risk (value of 5), and high risk (value of 10) on the basis of thresholds represented in the literature. For example, glycemic control, quantified by hemoglobin A1c (HbA1c) levels, are known to correlate with the outcomes of healing, and higher values are associated with poor wound healing [48]. Research [49] has shown that HbA1c levels above 8% indicate poor glycemic control, warranting the highest risk score of 10. Conversely, levels of 7–8% are regarded as fair glycemic control, with a medium risk score of 5; below 7% was given a low-risk score of 0. Through use of these thresholds, the A1c levels were normalized on a scale from 0 to 10.

In cases where only one threshold was reported in the literature to determine the risk, such as daily number of steps (above 3000 steps was defined to be high-risk [50,51]), we used an arbitrary selection based on the recommendations of clinical experts in our investigative team and a critical appraisal of the prior literature to define the risk level. For example, in the case of the daily number of steps, we assumed that fewer than 1000 steps per day would have a low risk, while more than 3000 steps would actually be considered a high risk. This decision was based on the findings reported in Jarl et al.’s systematic review [50], which noted that in users of both non-removable and removable walkers, the weekly reduction in the ulcer significantly and negatively correlated with the number of steps; each additional 1000 daily steps reduced the weekly healing rate (reduction in the ulcer’s area) by between 5% and 5.4%, depending on the type of offloading used. Similar results were reported in the study of Saltzman et al. [52]. 

For the outcomes of wound healing, on the basis of a critical appraisal of the prior literature and input from wound care specialists who referred patients to this study or were involved in their care, we defined three risk categories for wound healing as follows: (1) high risk (score of 10) or unfavorable healing, defined as a reduction in the wound’s area of less than 40% at 12 weeks compared with the baseline, along with adverse events (e.g., amputation, wound infection, hospitalization, death), or dropout, as recommended by Patry et al. [47]; (2) fair to good healing (score of 5), defined as a reduction in the wound’s area of 40% to less than 100% at 12 weeks compared with the baseline; and (3) closure of the wound (score of 0), defined as the full closure of wounds before or at 12 weeks. 

For adherence to the offloading device, we considered the consistency of using the offloading device and the number of steps taken with the offloading device together. Cases where the offloading device was used consistently (e.g., every day while active) and with less than 1000 steps taken with and without the offloading device were labelled as low-risk (score of 0), while the cases where the offloading device was used inconsistently and where the average number of steps taken with and without offloading device was more than 1000 but less than 3000 were labelled as medium-risk (score of 5), and cases where the offloading device was used inconsistently and more than 3000 steps were taken with and without the offloading device were labelled as high-risk (score of 10).

For metrics with no clear threshold recommended in the literature, we used the cohort and percentile approach to determine the low-, medium-, and high-risk groups. For example, for estimating exhaustion and slowness, we estimated, respectively, the percentage of the decrease in the elbow’s extension–flexion rotation speed, variability in the elbow’s extension–flexion speed, the average extension–flexion speed of the elbow, and the number of elbow flexions over the 20 s repetitive elbow flexion–extension test, as recommended in the study of Toosizadeh et al. [28]. Then the median values of the cohort for each parameter was then determined. Values lower than the medium value were labelled as low-risk (0), and those higher than the medium value were labelled as high-risk (10). 

**Figure 4:**
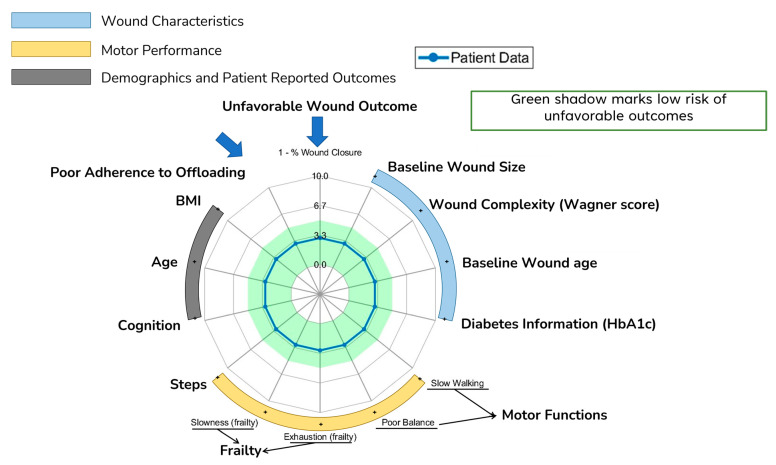
The holistic visualization system consists of a radar plot that displays digital metrics associated with the risk of unfavorable outcomes of the wound. Areas shaded in green indicate a low risk of unfavorable outcomes, offering a comprehensive visual representation of the patient’s data for medical assessment.

Table 2 details the specific thresholds used for categorizing these metrics as low-, medium-, or high risk. Radar charts based on these grades were created to provide a holistic visualization of the key digital biomarkers’ information by group, subgroup, and individual patients to the care providers. The radar chart was shaded in green for a low risk of unfavorable outcomes of the wound, where the grade was below the threshold. Figure 4 shows the radar chart and the green-shaded area. All analyses were performed using MATLAB R2022b (MathWorks, Natick, MA, USA) and SPSS 29.0 (IBM, Chicago, IL, USA); 0.05 was set as the level of statistical significance. 

## 3. Results

Of the 124 participants who met the inclusion criteria, 119 initially completed the study. However, 50 participants discontinued the study because of early dropouts for various reasons. These included an inability to attend the weekly clinic visits and the poor acceptability of offloading (21, 18%). Other reasons for discontinuation were adverse events, such as unplanned hospitalization, death, or limb amputation (4, 3%). In addition, seven of the remaining participants discontinued the study due to loss of eligibility, such as a lack of insurance or developing another disease. Consequently, data from the remaining 62 participants were deemed to be reliable and were utilized to develop and assess the remote visualization framework. This framework was designed to identify and display the digital biomarkers associated with poor wound healing. The participants’ demographics, the conditions of the foot ulcers at baseline, and unfavorable outcomes are shown in Table 3.

Among the 119 participants, 42% achieved successful healing, defined as ≥80% closure of the wound at 12 weeks. Meanwhile, 39% of the participants exhibited unfavorable outcomes, including less than 40% closure of the wound at 12 weeks or study-related discontinuation of the study. 

**Figure 5:**
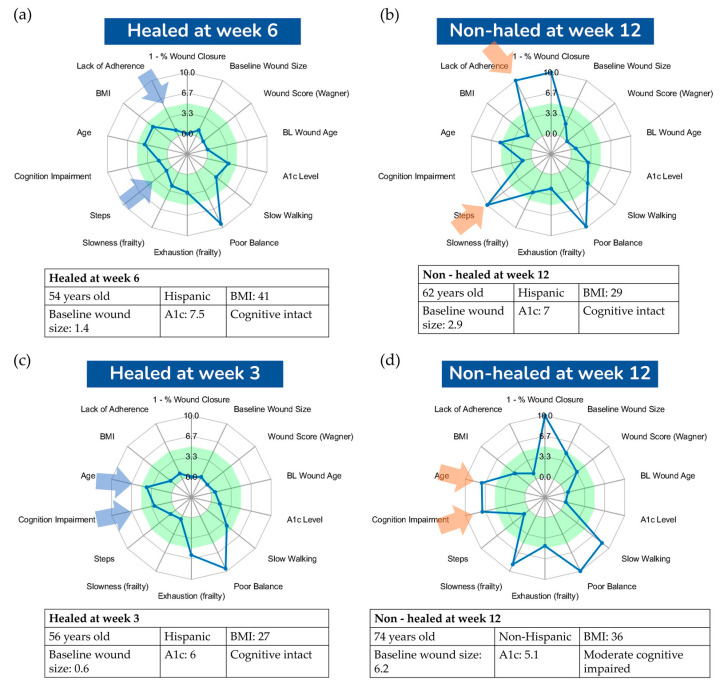
(**a**,**b**) Comparison of participants who healed at 6 weeks and those who did not heal at 12 weeks. Adherence level and number of steps were characteristics of poor healing. (**c**,**d**) Comparison of participants who healed at 3 weeks and those who did not at 12 weeks. Age and cognition were characteristics of poor healing.

Figure 5 presents a visualization comparing four DFU cases, all with well-controlled glycemia (A1c less than 7.5%); two did not heal by 12 weeks while the other two healed before the 12-week mark. This visualization aimed to offer deeper insights into the major risk factors influencing successful wound healing or contributing to failure. Both cases in Figure 5a,c healed before the 12-week threshold, sharing characteristics such as high adherence to offloading and a relatively low number of weight-bearing activities, as indicated by the small number of daily steps. In contrast, the case in Figure 5b did not heal by the 12-week mark, likely due to poor adherence to offloading and a high number of daily steps, presumably unprotected. This suggests that the combination of poor adherence and a high number of daily steps are key factors in suboptimal outcomes of wound healing. Interestingly, the case in Figure 5d demonstrated that good compliance with offloading and a low number of daily steps are not always sufficient for favorable outcomes of wound healing. We hypothesize that in scenarios where adherence is high and daily steps are low, other factors, such as cognitive function and frailty, could be significant risk factors for poor healing. This particular case had mild cognitive impairment and exhibited signs of frailty, such as slowness, exhaustion, and a low walking speed, potentially impacting the healing process. This aligns with other studies suggesting frailty as a determinant of the outcomes of wound healing [44,45]. We also hypothesize that in cases of poor adherence or a high number of daily steps, care providers could investigate the detailed daily summaries of adherence and daily steps, as illustrated in Figure 2. This would enable them to personalize education, such as discussing specific days or times of day when adherence to offloading is low or when patients have a significantly high number of steps. This may, in turn, assist in behavioral changes and the definition of an action plan. However, the effectiveness of these strategies in enhancing wound healing needs to be validated in future studies.

## 4. Discussion

This study aimed to design a holistic visualization tool to determine and visualize possible multiple digital biomarkers associated with poor diabetic wound healing. Our major findings suggest that using a holistic visualization system could help identify digital biomarkers associated with the unsuccessful healing of diabetic foot ulcers (DFUs) within a 12-week timeframe. By analyzing the radar plot for each patient, we were able to discern the combination of various parameters contributing to the healing outcomes. This approach allows for a comprehensive evaluation of multiple digital biomarkers, thereby enhancing our understanding of the factors that influence healing.

The utilization of a visualization system in this context has significant practical implications. Traditional methods for treating DFUs often rely on subjective assessments and limited data points, which can lead to suboptimal outcomes. However, our approach enables a more nuanced and personalized understanding of the healing process, thereby facilitating the development of more effective remote treatment plans. By considering a range of digital biomarkers, beyond adherence to offloading devices, we gained insights into the complex interplay of factors influencing the healing of DFUs. The implementation of our visualization system has the potential to revolutionize the management of DFUs. By providing clinicians with a clear and intuitive representation of digital biomarkers associated with healed and non-healed groups, the system enables informed decision-making. Clinicians can use this information to adjust treatment strategies, provide targeted interventions, and closely monitor patients’ progress. However, considering only the group effects can mask the individual parameters, which can be important for creating personalized treatment plans. For instance, within our sample group, the baseline wound size exhibited the most substantial effect size when distinguishing between the healed and non-healed groups. It is crucial to highlight that this parameter falls within the range that is indicative of a positive association with the healing process in our specific case studies. Conversely, other parameters such as age, cognition, and steps contributed to unsuccessful healing. Therefore, a thorough assessment of each case is imperative. This comprehensive evaluation would enable clinicians to identify and address the specific barriers to healing that may be present in individual patients. Consequently, personalized treatment plans can be tailored to meet specific needs, thereby enhancing the likelihood of successful outcomes.

Moreover, the use of remote treatment plans supported by visualization systems offers several advantages. This reduces the need for frequent in-person visits by providing convenient and efficient care planning. Patients can receive ongoing support and guidance, ensure adherence to treatment protocols, and facilitate early intervention in cases of poor adherence or other complications. This remote approach has the potential to significantly improve the patients’ outcomes, particularly in individuals who may face challenges in accessing healthcare facilities or adhering to traditional treatment regimens for personal or work-related reasons.

While this study was a sub-analysis of randomized controlled trial (RCT) studies examining the effectiveness of various offloading methods in enhancing the outcomes of wounds, the current presentation was observational in nature. The designed holistic visualization and its potential effectiveness in empowering care providers to personalize wound care and ultimately enhance the outcomes of wounds for patients with diabetic foot ulcers need to be validated in new RCTs and compared with the standard of care without the proposed visualization solution.

Although these preliminary findings are promising, our sample was both small and homogenous. This homogeneity might account for the lack of significant differences in the demographics and comorbidities between the healed and non-healed groups. Furthermore, most of the participants were male. Given these factors, there is a pressing need to validate our findings using a larger and more diverse sample that truly represents the demographic spread of individuals with DFUs. Additionally, in our study, to simplify the interpretation and visualization of all risk factors in a single holistic visualization graph, we normalized all parameters to a scale of 0 to 10 based on benchmarks of risk reported in the literature. However, for some of these benchmarks, the level of evidence to determine the level of risk may be limited, and, in some cases, we relied on the clinical expertise and our investigative team to determine healthy and risky benchmarks, such as the daily number of steps, for which we assumed that a number of steps less than 1000 had a low risk and a number of steps greater than 3000 was considered to be high-risk for wound healing. For some risk factors, such as exhaustion and weakness, since we could not identify a threshold based on values reported in the literature, we used the median value of the cohort to determine the threshold, which may be considered subjective and arbitrary. Therefore, future studies are warranted to validate our proposed benchmarks or to fine-tune them to better associate with poor wound healing in patients with DFUs. Furthermore, our proposed visualization solution may not include other potential risk factors that affect wound healing, such as adherence to the recommended diet and other measurable factors contributing to wound healing. These include vascular health, infection, the severity of neuropathy, nutritional deficiencies, the immune system’s function, lifestyle factors, and psychological stress. There was a risk of selection bias in this study, because for developing the visualization and normalization solution, we relied on available data including data collected from sensor-based offloading to collect daily steps, cadence, and adherence. In our study, several participants dropped out from the study, since they did not want to wear an offloading device, which is considered as standard of care, and some missed routine clinical visits, which affected the ability to track their wounds’ healing. Thus, the proposed visualization may have underestimated some of the potential risk factors, such as the effect of poor adherence or the effect of daily steps, as it limited the cohort to those with some level of compliance with wearing offloading devices. In addition, future studies could explore the long-term impact of the visualization system on the outcomes of healing with large clinical trials and investigate additional digital biomarkers that may further enhance our understanding of DFUs’ healing with the help of machine learning and deep learning algorithms. Additionally, assessing the cost-effectiveness and scalability of implementing such a system in a clinical setting in collaboration with stakeholders would be valuable.

## 5. Conclusions

In conclusion, our study suggests a holistic visualization system for identifying multiple digital biomarkers associated with successful healing of DFUs. By leveraging this approach, clinicians can gain valuable insights into the complex dynamics of healing, leading to personalized and effective treatment plans. Ultimately, the integration of such a system into routine clinical practice has the potential to significantly improve the management and outcomes of patients with DFUs.

## Figures and Tables

**Table 1:** Definitions of measurements.

Digital Biomarker	Definition	Measurement Tool
**Adherence to offloading device**		
Adherence (n.u.)	The consistency of the use of the offloading device and the number of steps taken with the boot and without the boot	Smart Boot, Sensoria Core [24]
**Characteristics of the wound**		
Baseline size of the wound (cm^2^)	Area of wound calculated by the wound monitoring device (eKare) at the baseline visit	Wound photos from eKare
Wound’s complexity (n.u.)	The complexity of the wound scored using Wagner	Medical chart [46]
Baseline age of the wound (days)	The time difference between the wound‘s onset date and the baseline date	Medical chart
HbA1c level (%)	Average blood sugar level over the past 3 months	Medical chart
**Motor Performance**		
Gait (steps/min) (*cadence*)	Number of steps taken per minute during TUG assessment; represents the motor function of the patient	Wearables, LegSys [34]
Balance (center of mass sway)	Patient’s ability to distribute their weight during the double- stance–eyes open assessment; represents the motor function of the patient	Wearables, BalanSense [35]
Exhaustion (frailty)	Decrease in the elbow’s extension–flexion speed, variability in the elbow‘s extension–flexion speed	Video-based 20 s arm flexion and extension exercise [28]
Slowness (frailty)	Average extension–flexion speed of the elbow, number of elbow flexions in 20 s	Video-based 20 s arm flexion and extension exercise [28]
Mobility (n) (*steps*)	Number of the steps taken during the daytime; represents the motor function of the patient	Smart Boot, Sensoria Core
**Demographics and** **p** **atient** **-r** **eported** **o** **utcomes**		
Cognition (score)	The mental action or process of acquiring knowledge and understanding, scored with MoCA assessment	12-point MoCA questionnaire [33]
Age (years)	Participant’s age	Medical chart
Body Mass Index (BMI) (kg/m^2^)	Patient’s weight in kilograms divided by the square of height in meters	Medical chart
**Outcome of the w** **ound**		
Healing (%)	Percentage of the reduction in the wound’s area each week	Wound photos from eKare [47]

**Table 2:** Thresholds of the digital biomarkers.

Digital Biomarker	Type of Value	Threshold
**Adherence to** **o** **ffloading** **d** **evice**		
Poor adherence	Categorical value	Low risk (0): use of the boot is consistent; step number is low
		Medium risk (5): use of the boot is inconsistent; step number is average
		High risk (10): use of the boot is inconsistent; step number is high
**Characteristics of the w** **ound**		
Baseline size of the wound	Continuous value	Low risk (0): ≤5 cm^2^
		High risk (10): ≥10 cm^2^
Wound’s complexity	Categorical value	Low risk (0): 0–1 Wagner score [53]
		Medium risk (5): 2–3 Wagner score
		High risk (10): 4–5 Wagner score
Baseline age of the wound	Continuous value	Low risk (0): ≤60 days before the baseline date [54]
		High risk (10): ≥240 days before the baseline date
A1c level	Continuous value	Low risk (0): ≤7% [49]
		High risk (10): ≥8%
**Motor** **p** **erformance**		
Slow walking	Continuous value	Low risk (0): ≥80 steps per minute [55,56]
		High risk (10): ≤60 steps per minute
Poor balance	Continuous value	Low risk (0): ≤0.5 center of mass sway
		High risk (10): ≥1,5 center of mass sway
Exhaustion (frailty)	Continuous value	Low risk (0): ≤ 0.12 normalized exhaustion score based on the cohort (median value)
		High risk (10): > 0.12 normalized exhaustion score based on the cohort (median value)
Slowness (frailty)	Continuous value	Low risk (0): ≤ 0.32 normalized slowness score based on the cohort (median value)
		High risk (10): > 0.32 normalized slowness score based on the cohort (median value)
Steps	Continuous value	Low risk (0): ≤1000 daily steps [51]
		High risk (10): ≥3000 daily steps
**Demographics and** **p** **atient** **-r** **eported** **o** **utcomes**		
Cognitive impairment	Continuous value	Low risk (0): ≥10 point out of 12 points [57]
		High risk (10): ≤6 points out of 12 points
Age	Continuous value	Low risk (0): ≤50 years [58]
		High risk (10): ≥65 years
Body Mass Index (BMI)	Continuous value	Low risk (0): ≤25 [59]
		High risk (10): ≥35
**Outcome of the** **Wound**		
Unfavorable outcome of the wound	Categorical value	Unfavorable healing (10): ≤40% wound closure [47]
		Fair to good healing (5): 40–100% wound closure
		Favorable healing (0): 100% wound closure

**Table 3:** Participants’ demographics and baseline conditions of the wounds.

Digital Biomarker	n = 124	
**Demographics**		
Age (years, median, (25–75th percentile))	57, (50, 65.5)	
Sex (male, %)	81%	
Ethnicity (Hispanic, %)	55%	
Race (%)	American Indian or Alaskan Native	2%
	Asian	4%
	Black or African American	6%
	Native Hawaiian or Pacific Islander	2%
	White	76%
	Other	1%
	No answer	10%
BMI (kg/m^2^, median, (25–75th percentile))	31, (27, 37)	
**Clinical characteristics**		
Area of the ulcer (cm^2^, median, (25–75th percentile))	1.3, (0.5, 3.2)	
Wagner score (n.u., median (25–75th percentile))	1, (1, 2)	
Wound age (days, median (25–75th percentile))	43, (4.8, 137.8)	
Ulcer’s location (forefoot, %)	52%	
Ulcer’s location (midfoot, %)	27%	
Ulcer’s location (hindfoot, %)	14%	
**Outcomes**	**n = 119**	
Favorable outcomes *, n (%)	50 (42%)	
Poor outcomes, n (%)	54 (45%)	
	Fair healing outcomes **, n, (%)	7 (6%)
	Not healed at 12 weeks ***, n (%)	5 (4%)
	Study related dropouts †, n (%)	42 (35%)
Non-study-related dropouts º		15 (13%)

* Favorable outcomes were defined as participants achieving a reduction in the wound’s area of 80% or more at 12 weeks compared with the baseline or achieving outcomes that made them suitable for surgical wound closure. ** Fair healing outcomes were defined as a reduction in the wound’s area between 40% and 80% at 12 weeks compared with the baseline. *** No healing outcome was defined as a reduction in the wound’s area of less than 40% at 12 weeks compared with the baseline. ^†^ Study-related dropout was defined as a major adverse event such as limb amputation, removal of consent after trying the offloading device, missing follow-ups, or discontinuation of the intervention. º Non-study-related dropout was defined as a loss of eligibility or screening failure, unrelated adverse events such as hospitalization due to COVID or heart issues, or loss of data due to technical issues.

## Data Availability

Data are available upon request by contacting the corresponding author.
